# Interactions with microbial consortia have variable effects in organic carbon and production of exometabolites among genotypes of *Populus trichocarpa*

**DOI:** 10.1002/pld3.544

**Published:** 2023-11-21

**Authors:** Alyssa A. Carrell, Miranda Clark, Sara Jawdy, Wellington Muchero, Gladys Alexandre, Jesse L. Labbé, Tomás A. Rush

**Affiliations:** ^1^ Biosciences Division Oak Ridge National Laboratory Oak Ridge Tennessee USA; ^2^ Department of Biochemistry and Cellular and Molecular Biology University of Tennessee‐Knoxville Knoxville Tennessee USA; ^3^ Present address: Technology Holding Salt Lake City Utah USA

**Keywords:** beneficial bacteria, metabolomics, mycorrhizal fungi, poplar exometabolites, total organic carbon

## Abstract

Poplar is a short‐rotation woody crop frequently studied for its significance as a sustainable bioenergy source. The successful establishment of a poplar plantation partially depends on its rhizosphere—a dynamic zone governed by complex interactions between plant roots and a plethora of commensal, mutualistic, symbiotic, or pathogenic microbes that shape plant fitness. In an exploratory endeavor, we investigated the effects of a consortium consisting of ectomycorrhizal fungi and a beneficial *Pseudomonas* sp. strain GM41 on plant growth (including height, stem girth, leaf, and root growth) and as well as growth rate over time, across four *Populus trichocarpa* genotypes. Additionally, we compared the level of total organic carbon and plant exometabolite profiles across different poplar genotypes in the presence of the microbial consortium. These data revealed no significant difference in plant growth parameters between the treatments and the control across four different poplar genotypes at 7 weeks post‐inoculation. However, total organic carbon and exometabolite profiles were significantly different between the genotypes and the treatments. These findings suggest that this microbial consortium has the potential to trigger early signaling responses in poplar, influencing its metabolism in ways crucial for later developmental processes and stress tolerance.

## 1 INTRODUCTION

Poplar is a short‐rotation woody crop that provides sustainable bioenergy. It is used as feedstock for multiple energy sources such as heat, electricity, and liquid transportation fuels (Georgiadis et al., 2017; Lemus & Lal, 2005; Sannigrahi et al., 2010). The poplar bioeconomy has focused their efforts on breeding programs with species that can perform phytoremediation, have large aerial biomass, and deep root systems, like *Populus trichocarpa* (Doty, 2008; Sannigrahi et al., 2010). More specifically, hybrids from *P. trichocarpa* and *P. deltoides* are desirable for their deep rooting systems and resistance to the fungal pathogens *Melampsora medusae* (Newcombe & Ostry, 2001) and *Sphaerulina musiva*, the causal agents of poplar rust and Septoria Leaf Spot and Stem Canker (Dunnell & Leboldus, 2017).

*P. trichocarpa* has a symbiotic relationship with ectomycorrhizal fungi, namely, *Laccaria bicolor*, and this association hinges on a susceptibility factor PtLecRLK1 (Tschaplinski et al., 2014). PtLecRLK1 is absent in *P. deltoides*, resulting in a lack of ectomycorrhizae with *L. bicolor* and thus differences in metabolic profiles of plant exudates (Labbé et al., 2019). In this same study, PtLecRLK1 transgenic lines were created in *Arabidopsis thaliana* ecotype Col‐0 to validate the PtLecRLK1 role in root colonization by the fungus in a non‐host. Then, the fungus ability to colonize roots was assessed. Additional plant physiology was analyzed such as differentially expressed genes through transcriptomic analysis and differentially expressed primary and secondary metabolites found in plant exudates by the wild‐type and 35S:PtLecRLK1 transgenic *A. thaliana*. 35S:PtLecRLK1 transgenic plant roots were shown to have a mantle and Hartig net indicating ectomycorrhizal symbiosis with *L. bicolor*. However, this symbiosis was not observed in the wild‐type. Moreover, there were significant differences in gene expression showing that the 35S:PtLecRLK1 transgenic plants had a downregulation in defense‐related genes compared with the wild‐type. These results correlated with the plant exudation results. Metabolites related to defense responses in plants were also downregulated in 35S:PtLecRLK1 transgenic plants when exposed to *L. bicolor* (Labbé et al., 2019).

Beneficial microbes (mutualists and symbionts) can enhance the access of a plant to soil nutrients and water while priming its immunity to resist biotic stresses (Averill et al., 2019; Bonfante & Genre, 2010; Clemmensen et al., 2013; Romano et al., 2020; Yu, Pieterse, et al., 2019). Mycorrhizal fungi and plant‐promoting bacteria are common microbes used as bioinoculants to promote plant growth and productivity because they often have synergistic effects (Santoyo et al., 2021). Bacterial and fungal communities within a poplar rhizosphere are shaped by the root exudates that include primary and secondary metabolites (Fracchia et al., 2021; Mangeot‐Peter et al., 2020; Veach et al., 2019). These findings prompted us to compare the effects of other beneficial microbes on the exudate composition of *P. trichocarpa* and to compare the influence of the genotypes on this trait.

In this study, we investigate the effects of three ectomycorrhizal (ECM) fungi and one bacterium species to synergistically benefit the development of *P. trichocarpa*. The microbes used for inoculation were selected because they are known to individually benefit or were isolated from *P. trichocarpa* or related species (Labbé et al., 2014; Tschaplinski et al., 2014; Vélez et al., 2021). *Cenococcum geophilum* (Dothideomycetes, Ascomycota) is a cosmopolitan ECM fungus that colonizes both *Populus* and *Pinus* (de Freitas Pereira et al., 2018; Peter et al., 2016; Vélez et al., 2021). *Hebeloma brunneifolium* (Hymenogastraceae, Basidiomycota) is an ECM fungus, found throughout the eastern USA (Hesler, 1977), and is predicted to be important in community assembly and function of the *Populus* microbiome (https://mycocosm.jgi.doe.gov/Hebvel2/Hebvel2.home.html). *L. bicolor* (Hydnangiaceae, Basidiomycota) is a cosmopolitan ECM fungal model species for genetic and system biology studies, particularly when colonizing *Populus* species (Martin et al., 2008). The endophytic bacterium, *Pseudomonas fluorescens* sp. strain GM41 (Pseudomonadaceae, Gammaproteobacteria), was collected from the roots of *Populus deltoides* (Brown et al., 2012). This bacterial strain harbors genes commonly found in endophytic bacteria isolated from *Populus* that are not always found in rhizosphere bacteria (Timm et al., 2015). A previous study has shown a positive interaction between *P. fluorescens* and *L. bicolor* resulting in increased radial growth and hyphal density of *L. bicolor*, alongside an increased number of secondary roots across multiple *Populus* genotypes (Labbé et al., 2014). These results suggest a tripartite mutualistic benefit between the two organisms and their *Populus* host (Labbé et al., 2014). GM41 was shown to have an increased colonization rate in both *P. deltoides* and *P. trichocarpa* compared with other strains of *P. fluorescens* previously collected from *Populus* (Labbé et al., 2014). Besides the ability of these beneficial organisms to colonize *Populus* roots and improve their growth in mono‐associations, there is evidence these microbes can be isolated from similar geographical locations in forest areas within the eastern USA (Brown et al., 2012; Di Battista et al., 1996; Hesler, 1977; Vélez et al., 2021) indicating they could have some interaction with each other in environments where species of *Populus* are grown.

This study aims to evaluate the effects of the microbial consortium inoculum on *P. trichocarpa* physiological and exudate profiles and whether those effects are conserved across different *P. trichocarpa* genotypes. Herein, we hypothesize that these microbes, when mixed in a consortium, could improve height, stem girth, and leaf development and change the profile of *P. trichocarpa* root exudate exometabolite biosynthesis, which could contribute to those promoting plant growth attributes. Previously published evidence had demonstrated that *C. geophilum*, *L. bicolor*, and *P. fluorescens* individually improve poplar growth and development (de Freitas Pereira et al., 2018; Labbé et al., 2014; Quoreshi & Khasa, 2008; Timm et al., 2015; Vélez et al., 2021). Along the same line, previous studies have shown species of *Hebeloma* isolated from poplar roots (Heslin & Douglas, 1986; Lacercat‐Didier et al., 2016) or field samples (Selle et al., 2005) can exhibit beneficial properties on poplar growth (Quoreshi & Khasa, 2008; Siemens & Zwiazek, 2008) or provide defenses against plant pathogens (Pfabel et al., 2012). However, the individual effects of *H. brunneifolium* on poplar growth and development is unknown and were not examined in this study. Results here demonstrate that when used in a mixed consortium, these microbes did not produce a synergistic effect on plant growth in four poplar genotypes. However, they caused changes in the root exudate exometabolomes which were plant host genotype dependent.

## 2 MATERIALS AND METHODS

### 2.1 Poplar genotypes used for this study

To assess the effect of beneficial microbes on *P. trichocarpa* physiology and metabolome, four different genotypes were used: BESC‐2, BESC‐286, BESC‐821, and SKWE 24‐4, which are part of the genome‐wide association study (GWAS) dataset from the BioEnergy Science Center (https://bioenergycenter.org/besc/gwas/). These genotypes were the optimal plant materials available at the initiation of the experiment and were also targets for future breeding efforts. Poplar genotypes were sent to Phenotype Screening Corporation (Knoxville, TN, U.S.A.) (https://www.phenotypescreening.com/) to conduct the experiments. For each genotype, we started with eight cuttings, then chose those of comparable size and root stage resulting in four biological replications for each treatment (microbial consortium or control) (32 plants in total). All propagules were received and processed on September 16, 2019. For rooting preparation, stems were trimmed to approximately 8″ in length, had at least one viable bud per stem, and cut both ends at angles. Stems that were discolored or had cold damage were discarded. Next, stems were soaked in 20% bleach solution for 18 min, triple‐rinsed with sterile water, the basal ends were dipped in root hormone solution (Hormodin), and then submerged in sterile water, covering 2/3 of the stem. The water depth was maintained at 2/3 height of the stem for 10 days until root development had occurred for transplantation.

### 2.2 Transplantation, environmental settings, and watering conditions

Transplantation and inoculation of microbial consortium occurred on September 26, 2019, into individual custom containers containing expanded polystyrene (EPS) beads as the growth substrate. The containers (45 × 200 × 1000 mm) made of fused‐EPS and the substrate beads (.5‐ to 1.2‐mm bead diameter) allow for non‐destructive, digital X‐ray imaging of the root system. A treatment of 1.1 ml of Tetra (algaecide) diluted in 50 L of water was introduced into the watering delivery system to prevent algae growth at the beginning of the experiment. A second algaecide treatment procedure was repeated after 3 weeks. The light intensity was ~300 μmol/m^2^/s (PAR), and the photoperiod gradually increased over several weeks to light‐condition the cutting (10 h from September 26 to 30, 2019; 12 h from October 1 to 7, 2019; and 14 h from October 7, 2019, to November 7, 2019). The watering rate was cycled on for 30 s (63 ml) and off for 270 s from September 26 to October 21, 2019. The watering rate was then reduced to 30 s “on” and 330 s “off” from October 21 to November 7, 2019, to reduce residual water in the substrate and improve root X‐ray image quality. The nutrient solution was a modified Hoagland's solution at 50% concentration. The laboratory air temperature varied between 19.5°C and 29.5°C, with a median of 23°C, whereas the relative humidity varied between 30% and 72%, with a median of 42% during the time of the experiment.

### 2.3 Beneficial microbial inoculum

Microbial consortium inoculum consisted of *L. bicolor* strain S238N, *C. geophilum* strain 1.58, and *H. brunneifolium* strain PMI1Jessy and *P. fluorescens* strain GM41 packaged together according to US Patent 20200245629A1 (https://patents.google.com/patent/US20200245629A1/en). Treated rooting stems were placed 10 in. into the pre‐wetted, with 50 ml of sterile water, EPS beads into individual containers. Three packages of the beneficial microbes were later placed 1 in. into the pre‐wetted EPS beads. Control plants followed the same preparation but consisted of empty packages. Transplants were manually watered and then switched to a drip irrigation system. Colonization rates or molecular analysis for the abundance of ectomycorrhizal fungi or the bacterium within root tissue was not evaluated in these experiments due to the destructive harvesting of plant tissue for physiology development and metabolite extractions.

### 2.4 Plant measurements observed

Plant physiology measurements were stem and sprout diameters, primary and secondary growth dry weights, new growth of shoots and roots, height, and vegetative growth rates, which are determined by their vegetative stage over time. The primary growth are the main branches from the stem, and secondary growth are new additional branches from the main branch. The secondary‐to‐primary growth ratio was calculated by dividing the dry weights of the secondary growth by the primary growth. The above‐ground traits were measured daily, whereas below‐ground traits were measured weekly through digital X‐ray images. The X‐ray images were then analyzed by their root size class, which is based on shape and distribution, through the software RhizoTraits version 1.0 (https://www.phenotypescreening.com/).

### 2.5 Carbon sequestration and metabolomic collection

In an exploratory effort to identify total organic carbon (TOC) and putative poplar metabolites produced in the presence of the microbial consortium, poplar exudates were collected as following: 7‐week post inoculation, the roots of the four intact plants of each treatment were incubated in 1 L of sterile water for 1 h. The root exudate suspensions were filtered through a .45‐mm filter, stored at −80°C, and then lyophilized. Samples were resuspended in doubled distilled water and subjected to dissolved carbon (total, inorganic, or organic) analysis by thermal decomposition carbon analysis on a Shimadzu® carbon/nitrogen analyzer at the Water Quality Core Facility at the University of Tennessee, Knoxville. TOC negative controls had an average concentration of −.56. Root exometabolites composition was determined through untargeted mass spectrometry at the Biological and Small Molecular Mass Spectrometry Core at the University of Tennessee (https://chem.utk.edu/facilities/biological-and-small-molecule-mass-spectrometry-core-bsmmsc/). The exometabolites were first separated by using a Hydro reverse‐phase (RP) high‐performance liquid chromatography (HPLC) followed by ionization by electrospray in negative mode on an Orbitrap mass spectrometer. Data were processed, and peaks were picked via MAVEN software v.2.0.3 (Clasquin et al., 2012; Melamud et al., 2010). Metabolite area counts were normalized to the dry sample weight and the TOC. The medium retention time, medium *m*/*z* values, compound ID, KEGG ID, and formula are listed in Table S1.

### 2.6 Statistical analyses

All statistical analyses were performed in R (4.5). A generalized linear model (GLM, Type III Chi‐square Wald test) was used to test the effects of beneficial microbial inoculation and *Populus* genotype. The microbial consortium treatment effect within genotypes was tested with Tukey's honest significant difference (HSD) with post hoc correction (rstatix .7.0). ⍺ = .05 was used to denote statistical significance in GLM and Tukey's HSD post hoc analyses. A heatmap of the log‐fold changes of metabolites from the beneficial microbe treated and control plants was generated to assess the differences in root exudates. The built‐in R *prcomp* and *autoplot* functions were used to perform principal component analysis (PCA) on metabolite abundances across treatments based on the average of two technical replications per genotype. Technical replications were used for the TOC analysis, PCA plot, and heatmap as the poplar roots were pooled together to identify secreted compounds between treatments and genotypes. Roots were pooled together to obtain the minimum detectable poplar compounds within the 1‐h incubation.

## 3 RESULTS AND DISCUSSION

### 3.1 Microbial inoculants showed no effect on plant growth parameters

Poplar growth rate, sprout diameter, height, dry biomass weights, and percentage of root growth varied across the poplar genotypes analyzed (*p* < .05) but were not significantly different in regards of the microbial inoculum (Table 1). Although the assessed plant growth parameters showed no significant differences in the presence and absence of the microbial inoculum, we speculate on three rationale that need to be further investigated. First, these beneficial microbes may not be strong mutualists of the genotypes examined in this study. In this regard, Johnson et al. (2012) explained that intraspecific diversity in poplar genotypes could promote the dominance of a particular fungal species under variable environments. Second, although these fungi and bacterium were isolated from fruiting bodies in proximity or directly from *P. trichocarpa*, they might not promote early poplar development under this experimental design, or they may not be effective when applied as a community. In this regard, a previous study has shown that when bacteria and fungi are applied to poplar roots, the bacterial colonization was dominant at the early time point (2 days), whereas the endophytic and ectomycorrhizal fungi were dominant at the later timepoint (50 days) (Fracchia et al., 2021). Lastly, these microbes may produce specialized secreted metabolites that might positively or negatively altering their microbial behavior (Ditengou et al., 2015; Labbé et al., 2014; Rush et al., 2020, 2022; Villalobos Solis et al., 2002) or mutualistic symbiosis with the host (Cope et al., 2019; Maillet et al., 2011). Because the focus of this study was on how these microbes influence poplar development, we did not investigate the effect that these microbes could have on each other when applied to a microcosm with and without a host.

**TABLE 1:** Linear model and *p* values for plant phenotypes.

	Genotype	Treatment	Treatment × genotype
Phenotype	*p* value	*p* value	*p* value
Diameter (mm)	.2317	.1760	.5079
Sprout diameter: cutting diameter	**.0043**	.6725	.9346
Sprout diameter (mm)	**.0002**	.1054	.4824
Dry weight (g) of primary growth	**.0002**	.0879	.3927
Dry weight (g) of secondary growth	**.0463**	.1897	.2130
Dry weight (g) of shoot	**.0015**	.0899	.3660
Dry weight (g) of root	**.0023**	.1304	.4673
New growth (g)	**.0016**	.0866	.3531
Height (mm)	.1364	.1763	.3963
Growth rate (mm/day)	.1364	.1763	.3963
Vegetative growth rate (mm/day)	**<.0001**	.8163	.2334
Ratio of secondary to primary growth	**<.0001**	.8189	.5432
Root (%)	**.0010**	.9186	.6297
Total organic carbon (ppm)	**<.0001**	**<.0001**	**<.0001**

*Note*: Bold numbers indicate signficant *p* values.

Several studies have independently inoculated these ectomycorrhizal fungi with a host and observed a mantle and Hartig net between 2 and 12 weeks (Courty et al., 2011; de Freitas Pereira et al., 2018; Labbé et al., 2014; Martin et al., 2008; Plett et al., 2014). In those studies, changes in gene expressions or enzymatic or secretum profiles of the fungus and the host were observed when the plant was colonized by ectomycorrhiza compared with the control. However, there were minimal to no data about the growth and development of the host. Therefore, the 7‐week period adapted in our study might be too short to observe any significant poplar growth and development changes.

### 3.2 Microbial inoculants impacted TOC content in plant roots

**FIGURE 1:**
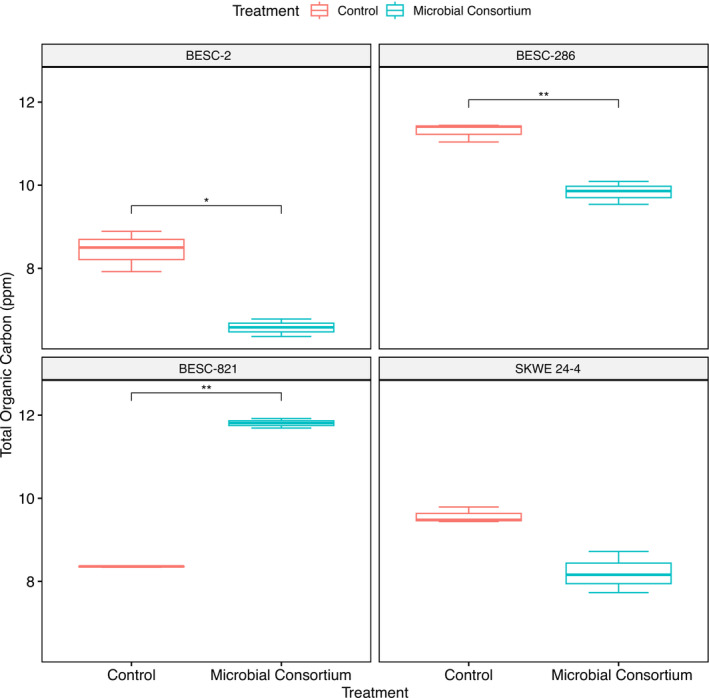
Total organic carbon measurements significantly differ between the control and plants inoculated with the microbial consortium in BESC genotypes. Linear model shows a *p* value <.001, where * is *p* value < .05, ** is *p* value < .01, and *** is *p* value < .001. Negative controls are not depicted but had a concentration of −.56. Data are based on three technical replications.

Overall, data from our study showed that TOC in plant roots was influenced by the addition of the microbes, but it was dependent on the poplar genotype (interaction treatment × genotype: *p* < .0001) (Figure 1, Table 1).

Significant values are in bold if the *p* value < .05. Plant phenotype data are based on biological replications (Table S2). TOC data are based on three technical replications.

The TOC was significantly higher in the exometabolites of inoculated *P. trichocarpa* genotype BESC‐821 (by 34.2%) compared with its non‐inoculated control. However, TOC was lower in *P. trichocarpa* genotypes BESC‐2 by 24.8% and BESC‐286 by 13.9% when compared with their controls. While the TOC of BESC‐2, BESC‐286, and BESC‐821 was significantly different, there was no significant difference in genotype SWKE 24‐4 between treatment and control. These findings may indicate that these three ECM fungi and bacterium are influential partners on *P. trichocarpa* by directly increasing organic carbon content in the rhizosphere with BESC‐821 but not BESC‐2, BESC‐286, and SWKE 24‐4. Mycorrhizal fungi have been found to stimulate carbon production in at least some plant hosts (Stuart & Plett, 2020). Alternatively, the difference in TOC might indicate a functional mycorrhizal symbiosis with mycorrhizal fungi utilizing carbon for growth and functioning (Frey, 2019; Jakobsen & Rosendahl, 1990) or indirectly effecting plant host carbon exudation through microbiome signaling altered root functional traits (Anderson & Cairney, 2007; Wen et al., 2019).

### 3.3 Microbial inoculants impacted the root exometabolite profiles

**FIGURE 2:**
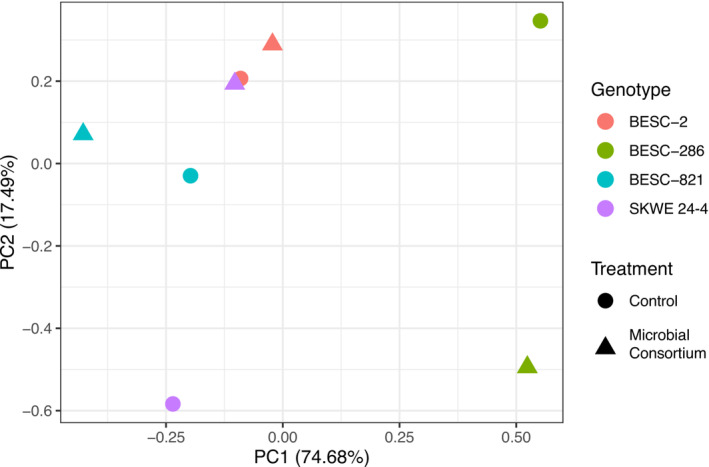
Principal component analysis (PCA) plot of pooled metabolites produced by poplar genotypes and the differences between the microbial consortium treatments and control. Data are based on pooled samples.

Because the microbial consortium showed a significant influence on the production of organic carbon from poplar, we were interested in exploring the potential impacts of this consortium on the root exometabolite profile. First, a PCA to determine the variance between genotypes and treated versus non‐treated samples showed variation between genotypes and their corresponding treated versus non‐treated samples. Two components accounted for 74.68% and 17.49% in variation with BESC‐286 samples distinct from the remaining genotypes (Figure 2). Interestingly, unlike the TOC results, there were variations between treatment and control in genotype SKWE 24‐4 for putative metabolites we had identified. These results are coherent with previous studies demonstrating early plant‐microbe interactions yield changes in the root secretum (Wong et al., 2019; Wong‐Bajracharya et al., 2020).

**FIGURE 3:**
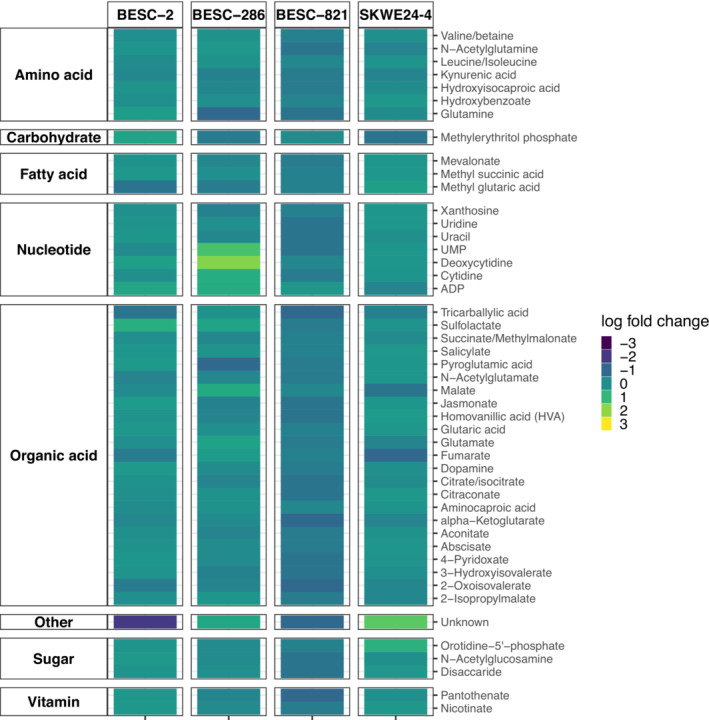
Global comparison of poplar exometabolites identified in the presence of the microbial consortium compared with the control samples. Data are based on pooled samples.

Next, we identified highly regulated exometabolites from pooled poplar‐treated samples and their control samples. Forty‐seven exometabolites were identified from the poplar genotypes in the presence of the microbial inoculum (Figure 3).

Overall, the microbial consortium may have stimulated the production of plant exometabolites for BESC‐2, BESC‐286, and SKWE 24–4, supporting part of our hypothesis. Although many of these metabolites identified are known to promote plant growth and development, it is too early to determine if those induced metabolites will have the same results with the different genotypes, as no differences in plant physiology were seen between treatments at 7 weeks. Additionally, possible changes in the abundance of metabolites measured could be due to microbial produced metabolites. This microbial consortium did not show the same effect with all tested genotypes because the same metabolites were downregulated in BESC‐821, suggesting there could be specificity from the host when interacting with microbes.

Herein, we highlight specific metabolites identified in our analysis and their potential roles. BESC‐2 treated samples showed a threefold decrease of an unidentified compound at a retention time 10.975 min and *m*/*z* fragment of 207.077 with the chemical formula C_10_H_12_N_2_O_3_. Interestingly, SWKE 24‐4 treated samples showed an opposite regulation of production of this same metabolite. There was a negative correlation between treated samples and the control for some notable metabolites like methyl glutaric acid, which plays a role in polymer production (Yu et al., 2022); tricarballylic acid, which interrupts the TCA cycle (Russell & Forsberg, 1986); and fumarate, which is predicted to be a carbon‐sink for photosynthate during nitrate assimilation (Araújo et al., 2011) in BESC‐2 treated samples. Conversely, there was a positive correlation of sulfolactate, a natural product in plants that can be degraded by microbes as a sole carbon and energy source for growth (Denger & Cook, 2010) in BESC‐2 treated samples. One could speculate that the microbial consortium is influencing the production of natural products for energy while negatively regulating other carbon‐sink sources.

BESC‐286 treated samples positively regulated nucleotide amounts stemming from the root system. For example, a 2.0‐to‐3.0‐fold increase in nucleotides like uridine monophosphate (UMP) and deoxycytidine was observed. UMP is an intermediate step in converting orotate into uridine (Zhang et al., 2020) and is involved in pyrimidine metabolism, biosynthesis of cofactors, and nucleotide metabolism. Deoxycytidine can improve cold tolerance in plants (Song et al., 2017) and is involved in nucleotide and pyrimidine metabolism, metabolic pathways, and ABC transporters. However, there was no significant impact on root growth in our system. There was downregulation of glutamine and pyroglutamic acid, which was expected because they are part of the same metabolic pathway of converting glutamine to pyroglutamic acid (Gowda et al., 2015). Glutamine plays a role in increased poplar growth and development, as shown through experiments with transgenic lines' overexpressing glutamine synthetase (Han et al., 2022; Man et al., 2011; Zhong et al., 2004). Pyroglutamic acid is an intermediate in glutathione metabolism, enhances photosynthesis and plant defense mechanisms in lettuce (Jiménez‐Arias et al., 2019), and is converted into glutamate in plants (Mazelis & Pratt, 1976). Perhaps the microbial consortium examined here could influence cold tolerance in this genotype or influence nitrogen metabolism due to the glutamate/glutamine interplay with oxoglutarate for amino acid synthesis coordinated with TCA cycle activity (Luo et al., 2013). Lastly, methylerythritol phosphate and mevalonate were downregulated and critical in plant terpene pathways (Martineau et al., 2010; Movahedi et al., 2021). It is noteworthy that both BESC‐2 and BESC‐286 treated samples decreased TOC but increased several exometabolites detected from the plant roots, indicating there are unknown carbon sources that were downregulated and detected in the TOC analysis.

Most metabolites detected in BESC‐821 treated samples were nearly half as abundant as compared with the control in all categories, notably in organic acids, which are critical for soil nutrient availability (Adeleke et al., 2017); however, we observed an increase of TOC in the presence of the microbial consortium (Figure 1). The TOC analyses measure total carbon in the samples, whereas the metabolic profiling only identifies known compounds in the library used to identify spectral signatures. These results suggest that most of the total carbon contributing to TOC correspond to unknown metabolites. Because root exudates are composed of amino acids, enzymes, organic acids, sugars, and vitamins (Adeleke et al., 2017), the microbial consortium interferes more with BESC‐821 metabolomic pathway responses in the rhizosphere than the other genotypes examined. These results could indicate that the BESC‐821 defenses are lowered in the presence of these microbes. However, it could also indicate that BESC‐821 and these microbes are not good biological partners because a previous study has shown that the downregulation of soluble sugars, organic acids, and amino acids coupled with the upregulation of GABA (not tested in this study) had inhibited adventitious roots, critical for vegetative propagation (van der Merwe et al., 2009; Yue et al., 2018). Yet, no differences in root morphology or biomass were observed in this study between the treated and control BESC‐821. Another consideration is that several downregulated metabolites belong to the shikimate‐phenylpropanoid pathway, which is essential for plants' primary metabolism and aromatic amino acids' biosynthesis (Movahedi et al., 2021). Notably, there was a downregulation of plant hormones like abscisate (also known as abscisin II—PubChem ID 7251168). This plant hormone is an abscisic acid that can regulate drought adaptation in plants and, more specifically, balances biomass production and climate adaptation in poplar (Yu, Wildhagen, et al., 2019).

SKWE 24‐4 treated samples had a notable decrease in methylerythritol phosphate, fumarate, and malate. Methylerythritol phosphate is predicted to regulate isoprenoid biosynthesis (Frank & Groll, 2017), which are secondary metabolites vital for plant growth and development, membrane fluidity, photosynthesis, and respiration (Movahedi et al., 2022). Fumarate and malate are both central TCA cycle metabolites linked together. Whenever fumarase is present, an enzyme catalyzes its fumarate's hydration to malate (Gajewski et al., 1985). Malate plays a role in plant defense (Casati et al., 1999), nutrition (Schulze et al., 2002), and starch metabolism (Centeno et al., 2011). Lastly, two putative metabolites were upregulated, which were orotidine‐5′‐phosphate, which is the last intermediate step for biosynthesis of UMP, and an unknown compound. These results suggest the microbial consortium downregulates poplar's defense mechanisms while influencing the UMP synthesis, which did not have an observed effect on plant phenotypes.

## 4 CONCLUSION

In this study, we demonstrated that ectomycorrhizal fungi and *Pseudomonas* sp. strain GM41 influence poplar's ability to regulate TOC and exometabolite production in roots. However, this regulation was not conserved across different *P. trichocarpa* genotypes. To our knowledge, no previous studies have explored the differences between various poplar genotypes in response to microbial inoculants. Although our study highlighted the production of certain exometabolites known to contribute to plant growth and development, no phenotypic differences were observed on these plants after 7‐week incubation period. Future studies are required to assess whether the microbial consortia impact poplar phenotypes and how the differential metabolomic patterns relate to potential phenotypic differences through extended field study experiments. Interestingly, previous studies have identified poplar metabolites expressed in the presence of microbial pathogens (Movahedi et al., 2021); however, few of the reported compounds were identified in our study. These results could indicate a unique metabolomic response from poplar in response to beneficial compared with pathogenic microbes (Zeilinger et al., 2016).

## AUTHOR CONTRIBUTIONS

J.L.L. created the experimental design. M.C., S.J., and W.M. provided poplar cuttings for the experiment. G.A. prepared samples for mass spectrometry and total organic carbon analysis. A.A.C. and T.A.R. performed the statistical analyses and wrote the manuscript with the contribution of all coauthors.

## CONFLICT OF INTEREST STATEMENT

The authors declare no competing interests.

## PEER REVIEW

The peer review history for this article is available in the Supporting Information for this article.

## Supporting information


**Data S1** Supporting Information.

**Data S2** Supporting Information.

**Data S3** Supporting Information.

## Data Availability

LC/MS data are available through the MassIVE repository https://massive.ucsd.edu/ProteoSAFe/static/massive.jsp, Dataset Id: MSV000090574, Password: POPLAR, ftp://massive.ucsd.edu/MSV000090574/ and MetaboLights (MTBLS1727).
