# Supplementary material for: Interactions with microbial consortia have variable effects in organic carbon and production of exometabolites among genotypes of Populus trichocarpa
Source: Plant Direct. 2023 Nov 21;7(11):e544. doi: 10.1002/pld3.544 (PMC10660807; doi:10.1002/pld3.544)
Supplement: Supplementary file 3 — Data S3 Supporting Information. [file PLD3-7-e544-s003.pdf]

Plant Direct
Open Access

Interactions with microbial consortia have variable effects in organic carbon and production of exometabolites among genotypes of *Populus trichocarpa*

Alyssa A. Carrell, Miranda Clark, Sara Jawdy, Wellington Muchero, Gladys Alexandre, Jesse L. Labbé, Tomás A. Rush

DECISION LETTER – ROUND 1

July 13, 2023

Dr. Tomas Allen Rush
Oak Ridge National Laboratory
Bioscience Divsion
1 Bethel Valley Road
Oak Ridge, Tennessee 37831-2008

RE: Interactions with microbial consortia have variable effects in organic carbon and production of exometabolites among genotypes of Populus trichocarpa

Dear Dr. Rush:

Thank you for submitting to Plant Direct. All required reviews have been returned and we have now finished our evaluation of your manuscript. In light of the reviewers' and editor's comments, further revisions are needed before the paper can be accepted for publication in Plant Direct.

Please view the editors' and reviewers' comments below and use their suggestions as a guide while you work on your revision.

When uploading the revised version of this article, please be sure to include the following:

-A word document that contains your response to the reviewers. You should respond to each reviewer comment and note the changes made to the manuscript. If you do not agree with a reviewer's comment and choose not to make a suggested revision, please explain why. Please try to provide as complete an answer as possible to each reviewer's criticisms.
-A tracked changes document with each change highlighted
- A clean version of the latest version of the manuscript

I agree with the reviewers that the manuscript will be acceptable for publication after revisions. I

would like to highlight to 1) add additional details to the methods section and 2) make sure the claims stated are fully supported by the results (e.g. limitations in experimental design may limit the interpretation of results).

To upload your revision, please click the link below.
https://plantdirect.msubmit.net/cgi-bin/main.plex?el=A7Lr5bD3A3oFY3I3A9ftdOgZBsXyLIS0knkasV5B4AZ

In order to provide as timely a service as possible, we ask that your revision is resubmitted within three months after receipt of this request. If an extension is needed, please send a request, along with a brief explanation, to the editorial office at plantdirect@wiley.com .

Please note that, in addition to publishing reviewer comments, the author's responses to review comments will also be published alongside the final version of the paper. If you would not like the author's responses to be published, please contact the editorial office at plantdirect@wiley.com .

Thank you very much for giving us an opportunity to review your work. I look forward to receiving the next version.

Sincerely,

Hannah Schneider

Gustavo MacIntosh

Editor, Plant Direct

--- Editor comments:
I agree with the reviewers that the manuscript will be acceptable for publication after revisions. I would like to highlight to 1) add additional details to the methods section and 2) make sure the claims stated are fully supported by the results (e.g. limitations in experimental design may limit the interpretation of results).

--- Reviewer comments:
Reviewer #1:

The authors investigate morphological and metabolic changes in poplar after inoculation with a consortium of three ectomycorrhizal fungi and one plant-growth promoting bacterium, compared to noninoculated controls. They find no changes in morphology, but discuss alterations in the metabolome. The study presented presents an interesting idea, and the morphological studies are well-conducted. However, with the metabolic analyses, there are severe limitations due to the experimental design. Specifically, all biological replicates of the different genotypes were pooled into one (due to low metabolite concentrations), which allows no judgment of within-pool variation. Thus, no clear conclusions can be drawn regarding differences between genotypes, or between

inoculated vs noninoculated treatments. The limitations of the experimental design must be stated and discussed more clearly than they are at present. Special attention needs to be given to avoid statements of 'statistically significant differences' between treatments or genotypes. At most, the authors can discuss trends observed. One additional strong limitation of the study is the missing control of inoculation efficiency overall, and of the single strains separately.

remark to the abstract: reading the abstract first, I would include the treatment time of poplar with the microbiome, as an obvious limitation regarding evaluation of poplar morphology might be time (treatment too short to see the effect reflected in morphology).

L43: I did not look up the primary literature, but from the phrasing it is unclear to me if the authors transformed RLK{section sign} into P. trichocarpa or P. deltoides?
L44: is the differential regulation of exudation in colonized wt P. deltoides vs colonized 35S:RLK? please specify.
L115: in what were the beads soaked?
L115: do I picture correctly that the plants were grown in pots filled with PSC beads? Is this similar to a hydroponic setup? What size were the containers?
L118: why the change in light duration?
L120: what was the light intensity? was humidity controlled?
L136: please specify primary and secondary growth?
L197: did the authors observe a Hartig net or any other indication for ectomycorrhiza colonization when collecting the samples?
L212: unclear statement, is this comparing inoculated vs noninoculated?
L220: I disagree with the authors interpretation. The lower TOC in exudates might indicate a functional mycorrhizal symbiosis ongoing (mycorrhiza are provided with up to 20% of carbohydrates), and it was shown that mycorrhized plants have lower C exudation levels.
L223: again there is another interpretation: maybe TOC values are increased due to some indirect effect, maybe altered root morphology, or a microbiome signaling effect. typically, mycorrhiza decrease C exudation. For increased C exudation, photosynthesis rate would need to go up, which was not evaluated in this study. These speculations should go in the discussion.
L232: I find it really hard to discuss differences between treatments and genotypes in the PCA plot because there are no replicates. The authors do not know if they had analyzed all plants separately, if they overlapped completely. In my opinion, no conclusions can be drawn from this. Maybe it can be stated that 286 is distinct from the other lines because it clusters far apart, and most of the variation is explained by that.
L252: which statistical test was made to show stimulation, with one replicate only?
L264: do you have a putative ID for this metabolite, e.g. with a search of the exact mass?
L260: the authors should mention that an increased abundance of compounds could also be due to microbial production. Also, as the authors compare single values of treated vs control to each other with no means of judging biological variance within the dataset, it has to be very clearly mentioned here that all you can do is discuss trends.
L273: imprecise. An upregulation in nucleotide exudation does not necessarily coincide with elevated root levels. levels could be up in roots and shoots, or simply biosynthesis and transport could be upregulated with increased exudation but similar tissue levels.
L294: do you mean most metabolites were half as abundant in treated vs control?
L304: JA is typically involved in defense against herbivores, not microbes. there you would look into salicylic acid.

Figures: it needs to be stated in all legends that this data is based on pooled data or on technical replicates (TOC).
Figure 1: please indicate number of replicates, and please highlight that these are technical replicates.
Figure 1: please add TOC measurements of negative controls. How much above background are the measurements?
Table 1: please provide the raw values of the phenotypic data. I am specicifally interested in seeing how much root mass was there.

DECISION LETTER- ROUND 2

October 10, 2023

Dr. Tomas Allen Rush
Oak Ridge National Laboratory
Bioscience Divsion
1 Bethel Valley Road
Oak Ridge, Tennessee 37831-2008

RE: Interactions with microbial consortia have variable effects in organic carbon and production of exometabolites among genotypes of Populus trichocarpa

Dear Dr. Rush:

Thank you for submitting to Plant Direct. All required reviews have been returned and we have now finished our evaluation of your manuscript. In light of the reviewers' and editor's comments, further revisions are needed before the paper can be accepted for publication in Plant Direct.

Please view the editors' and reviewers' comments below and use their suggestions as a guide while you work on your revision.

When uploading the revised version of this article, please be sure to include the following:

-A word document that contains your response to the reviewers. You should respond to each reviewer comment and note the changes made to the manuscript. If you do not agree with a reviewer's comment and choose not to make a suggested revision, please explain why. Please try to provide as complete an answer as possible to each reviewer's criticisms.
-A tracked changes document with each change highlighted
- A clean version of the latest version of the manuscript

Thank you for your revisions. Please consider making some statements and conclusions in the manuscript a bit more subtle to reflect the limited number of genotypes and replications in the study. For example, in the abstract line 23 "across various poplar genotypes" could be changed to

'across four different poplar genotypes' and line 107-108 'on plant growth. However they did produce changes in the root...' could be changed to 'on plant growth in four poplar genotypes. However, they did produce changes in the root exudate exometabolomes, which results suggested was plant host genotype dependent'.

To upload your revision, please click the link below.
https://plantdirect.msubmit.net/cgi-bin/main.plex?el=A1Lr3bD2B7oFY2I3A9ftdOgZBsXyLIS0knkasV5B4AZ

In order to provide as timely a service as possible, we ask that your revision is resubmitted within three months after receipt of this request. If an extension is needed, please send a request, along with a brief explanation, to the editorial office at plantdirect@wiley.com .

Please note that, in addition to publishing reviewer comments, the author's responses to review comments will also be published alongside the final version of the paper. If you would not like the author's responses to be published, please contact the editorial office at plantdirect@wiley.com .

Thank you very much for giving us an opportunity to review your work. I look forward to receiving the next version.

Sincerely,

Hannah Schneider

Gustavo MacIntosh

Editor, Plant Direct

-- Editor comments:
Thank you for your revisions. Please consider making some statements and conclusions in the manuscript a bit more subtle to reflect the limited number of genotypes and replications in the study. For example, in the abstract line 23 "across various poplar genotypes" could be changed to 'across four different poplar genotypes' and line 107-108 'on plant growth. However they did produce changes in the root...' could be changed to 'on plant growth in four poplar genotypes. However, they did produce changes in the root exudate exometabolomes, which results suggested was plant host genotype dependent'.

-- Reviewer comments:
Reviewer #1:

thank you for your responses.

AUTHORS' RESPONSE

We are very grateful for the time that the reviewer spent reading our manuscript and the very valuable comments and suggestions. We have carefully considered the recommendations and addressed all of them to improve the manuscript.

Please find below a detailed point-by-point response to all comments. The reviewer comments are in black, and our responses are in blue. We also provide a revised version of the manuscript in which all modifications have been highlighted ("Carrell et al, - Manuscript Revised").

Please note that the line numbers that we refer to below are for the "Carrell et al, - Manuscript Revised."

Reviewer #1:

The authors investigate morphological and metabolic changes in poplar after inoculation with a consortium of three ectomycorrhizal fungi and one plant-growth promoting bacterium, compared to noninoculated controls. They find no changes in morphology, but discuss alterations in the metabolome. The study presented presents an interesting idea, and the morphological studies are well-conducted. However, with the metabolic analyses, there are severe limitations due to the experimental design. Specifically, all biological replicates of the different genotypes were pooled into one (due to low metabolite concentrations), which allows no judgment of within-pool variation. Thus, no clear conclusions can be drawn regarding differences between genotypes, or between inoculated vs noninoculated treatments. The limitations of the experimental design must be stated and discussed more clearly than they are at present. Special attention needs to be given to avoid statements of 'statistically significant differences' between treatments or genotypes. At most, the authors can discuss trends observed. One additional strong limitation of the study is the missing control of inoculation efficiency overall, and of the single strains separately.

We acknowledge that the pooling of data for the metabolomic results has limitations. Before continuing to a costly and large-scale project, this study aimed to identify any differences in the total organic carbon or poplar metabolomic profiles when inoculated with the microbes. Since no publications showed results for this idea about how a consortium of microbes would influence different poplar genotypes, we determined these initial results warrant a manuscript suitable for publication. Given our results in this study, we are pursuing a large-scale study with biological replications with selected poplar genotypes. Lastly, we had made clarifications and grammar corrections throughout the manuscript.

remark to the abstract: reading the abstract first, I would include the treatment time of poplar with the microbiome, as an obvious limitation regarding evaluation of poplar morphology might be time (treatment too short to see the effect reflected in morphology).

We agree with the reviewer; the time period is included in lines 21-23. " These data revealed no significant difference in plant growth parameters between the treatments and the control across the various poplar genotypes at seven weeks post-inoculation."

L43: I did not look up the primary literature, but from the phrasing it is unclear to me if the authors transformed RLK{section sign} into P. trichocarpa or P. deltoides?

Sorry for the lack of clarity. Labbe et al., (2019) made transgenic lines in *Arabidopsis thaliana*, not *Populus*. Line 44 “PtLecRLK1 transgenic lines were created in *Arabidopsis thaliana* ecotype Col-0”

L44: is the differential regulation of exudation in colonized wt P. deltoides vs colonized 35S:RLK? please specify.

The differential regulation of exudation was between wild-type *A. thaliana* and 35S:PtLecRLK1 transgenic *A. thaliana* after they were colonized by *Laccaria bicolor.* We restructured Lines 40 to 55 for a better clarification of the Labbe et al., 2019 study.

Lines 40 to 55: *Populus trichocarpa* has a symbiotic relationship with ectomycorrhizal fungi, namely *Laccaria bicolor*, and this association hinges on a susceptibility factor PtLecRLK1 (Tschaplinski et al., 2014). PtLecRLK1 is absent in *P. deltoides*, resulting in a lack of ectomycorrhizae with *L. bicolor* and thus differences in metabolic profiles of plant exudates (Labbé et al., 2019). In this same study, PtLecRLK1 transgenic lines were created in *Arabidopsis thaliana* ecotype Col-0 to validate the PtLecRLK1 role in root colonization by the fungus in a non-host. Then, the fungus ability to colonize roots were assessed. Additional plant physiology was analyzed such as differentially expressed genes through transcriptomic analysis and differentially expressed primary and secondary metabolites found in plant exudates by the wild-type and 35S:PtLecRLK1 transgenic *A. thaliana.* 35S:PtLecRLK1 transgenic plant roots were shown to have a mantle and Hartig net indicating ectomycorrhizal symbiosis with *L. bicolor.* However, this symbiosis was not observed in the wild-type. Moreover, there were significant differences in gene expression showing that the 35S:PtLecRLK1 transgenic plants had a down-regulation in defense-related genes compared to the wild-type. These results correlated with the plant exudation results. Metabolites related to defense responses in plants were also down-regulated in 35S:PtLecRLK1 transgenic plants when exposed to *L. bicolor* (Labbé et al., 2019).

L115: in what were the beads soaked?

Our apologies; the beads were not pre-soaked. The plant was placed into the expanded polystyrene (EPS) beads. Then a treatment of 1.1 ml of Tetra diluted in 50L of water was introduced into the watering delivery system at the beginning of the experiment. Please see lines 126 to 133 and 136 to 139.

Lines 126 to 133: Transplantation and inoculation of microbial consortium occurred on September 26, 2019, into individual custom containers containing expanded polystyrene (EPS) beads as the growth substrate. The containers (45mm x 200mm x 1000mm) made of fused-EPS and the substrate beads (0.5mm – 1.2mm bead diameter) allow for non-destructive, digital X-ray imaging of the root system. A treatment of 1.1 ml of Tetra (algaecide) diluted in 50 L of water was introduced into the watering delivery system to prevent algae growth at the beginning of the experiment. A second algaecide treatment procedure was repeated after three weeks.

Lines 136 to 139: The watering rate was cycled on for 30 seconds (63 ml) and off for 270 seconds from September 26 to October 21, 2019. The watering rate was then reduced to 30 seconds “on” and 330 seconds “off” from October 21 to November 7, 2019, to reduce residual water in the substrate and improve root X-ray image quality.

L115: do I picture correctly that the plants were grown in pots filled with PSC beads? Is this similar to a hydroponic setup? What size were the containers?

Yes, you are correct. The plants were grown in customized containers containing the EPS beads, and we had a water delivery system established. However, our setup is semi-hydroponic and mimics soil in pot conditions. The water is not drained out of the containers, and the EPS beads are used to stabilize the root system, like soil. In addition, we used the EPS beads so we could take digital X-ray images of the roots. Please see lines 126 to 143 for a detailed explanation.

L118: why the change in light duration?

We had the change in light duration to accustom the plantlets to the intensity of the heat from the lights.

L120: what was the light intensity? was humidity controlled?

Please see lines 133 to 136: The light intensity was ~300 micromoles/m^2/s (PAR), and the photoperiod gradually increased over several weeks to light-condition the cutting (10 hours from September 26 to 30, 2019; 12 hours from October 1 to 7, 2019; and 14 hours from October 7, 2019, to November 7, 2019).

Humidity was not controlled, but it was monitored. Lines 140 to 143: The laboratory air temperature varied between 19.5 °C and 29.5 °C, with a median of 23°C, while the relative humidity varied between 30% and 72%, with a median of 42% during the time of the experiment.

L136: please specify primary and secondary growth?

We added lines 155 to 163 to better specify primary and secondary growth.

"Plant physiology measurements were stem and sprout diameters, primary and secondary growth dry weights, new growth of shoots and roots, height, and vegetative growth rates, which is determined by their vegetative stage over time. The primary growth are the main branches from the stem, and secondary growth are new additional branches off of the main branch. The secondary-to-primary growth ratio was calculated by dividing the dry weights of the secondary growth by the primary growth."

L197: did the authors observe a Hartig net or any other indication for ectomycorrhiza colonization when collecting the samples?

In previous studies, a mantle and Hartig net were observed, as mentioned in Lines 217 to 219. "Several studies have independently inoculated these ectomycorrhizal fungi with a host and observed a mantle and Hartig net between 2 to 12 weeks (Martin et al., 2008; Courty et al., 2011; Labbé et al., 2014; Plett et al., 2014; de Freitas Pereira et al., 2018)."

However, our study focused on the plant and not the fungus' ability to colonize the roots. Fungi and bacteria are known to influence plants through both contact and non-contact ways. We explained on lines 152 to 154, that we did not look like colonization of the microbes on the roots. "Colonization rates or molecular analysis for the abundance of ectomycorrhizal fungi or the bacterium within root tissue were not evaluated in these experiments due to the destructive harvesting of plant tissue for physiology development and metabolite extractions."

L212: unclear statement, is this comparing inoculated vs noninoculated?

Please see revised lines 232 to 233, "The TOC were significantly higher in the exometabolites of inoculated *P. trichocarpa* genotype BESC-821 (by 34.2%) compared to its non-inoculated control."

L220: I disagree with the authors interpretation. The lower TOC in exudates might indicate a functional mycorrhizal symbiosis ongoing (mycorrhiza are provided with up to 20% of carbohydrates), and it was shown that mycorrhized plants have lower C exudation levels.

We thank the reviewer for the alternative interpretation and have included the below text in the manuscript (Lines 240 to 243):

"Alternatively, the difference in TOC might indicate a functional mycorrhizal symbiosis with mycorrhizal fungi utilizing carbon for growth and functioning (Jakobsen and Rosendahl, 1990) or indirectly effecting plant host carbon exudation through microbiome signaling, altered root functional traits (Wen et al., 2019). "

L223: again there is another interpretation: maybe TOC values are increased due to some indirect effect, maybe altered root morphology, or a microbiome signaling effect. typically, mycorrhiza decrease C exudation. For increased C exudation, photosynthesis rate would need to go up, which was not evaluated in this study. These speculations should go in the discussion.

As described above, we have added an alternative interpretation to our text. Again, we sincerely appreciate the reviewer's suggestion (Lines 240 to 243).

L232: I find it really hard to discuss differences between treatments and genotypes in the PCA plot because there are no replicates. The authors do not know if they had analyzed all plants separately, if they overlapped completely. In my opinion, no conclusions can be drawn from this. Maybe it can be stated that 286 is distinct from the other lines because it clusters far apart, and most of the variation is explained by that.

The PCA depicts the relationship of each pooled sample we analyzed. While there may be high biological variation not captured here, we see clustering based on genotype, especially for BESC-286, as the reviewer mentioned. We have updated the text to describe the distinct clustering of the genotype BESC-286 (Lines 255 to 258).

“Two components accounted for 74.68% and 17.49% in variation with BESC-286 samples distinct from the remaining genotypes (**Fig. 2**). Interestingly, unlike the TOC results, there were variations between treatment and control in genotype SKWE 24-4 for putative metabolites we had identified.”

L252: which statistical test was made to show stimulation, with one replicate only?

We did not provide a statistical test on the metabolites because we did not have biological replication. We have softened our language to describe a potential stimulation rather than a distinct one since we lack statistical support (Line 265 to 266).

“Next, we identified highly regulated exometabolites from pooled poplar-treated samples and their control samples.”

L264: do you have a putative ID for this metabolite, e.g. with a search of the exact mass?

There were multiple putative IDs for this compound. Without doing fragmentation patterns or comparison to a commercial standard, we did not believe it would be appropriate to speculate what the compound could be for this manuscript. However, the identity of this compound is the subject of a different manuscript we are drafting.

L260: the authors should mention that an increased abundance of compounds could also be due to microbial production. Also, as the authors compare single values of treated vs control to each other with no means of judging biological variance within the dataset, it has to be very clearly mentioned here that all you can do is discuss trends.

We agree with the reviewer and have updated the manuscript to indicate our comparisons are of pooled samples rather than individually as biological replications (Lines 276 to 277).

"Additionally, it is possible changes in the abundance of metabolites measured could be due to measured microbial produced metabolites."

L273: imprecise. An upregulation in nucleotide exudation does not necessarily coincide with elevated root levels. levels could be up in roots and shoots, or simply biosynthesis and transport could be upregulated with increased exudation but similar tissue levels.

We agreed with the reviewer, and we revised the sentence. We did not mean that the upregulation of nucleotide exudation resulted in elevated root levels. We did not see elevated root levels, but the exudation did come from the roots.

We revised lines 293 to 299 as "BESC-286 treated samples positively regulated nucleotide amounts stemming from the root system. For example, a 2.0-to-3.0-fold increase in nucleotides like uridine monophosphate (UMP) and deoxycytidine was observed. UMP is an intermediate step in converting orotate into uridine (Zhang et al., 2020) and is involved in pyrimidine metabolism, biosynthesis of cofactors and nucleotide metabolism. Deoxycytidine can improve cold tolerance in plants (Song et al., 2017) and is involved in nucleotide and pyrimidine metabolism, metabolic pathways, and ABC transporters. However, there was no significant impact on root growth in our system."

L294: do you mean most metabolites were half as abundant in treated vs control?

We agree with the reviewer and revised Lines 314 to 315: "Most metabolites detected in BESC-821 treated samples were nearly half as abundant as compared to the control in all categories, notably in organic acids, which are critical for soil nutrient availability (Adeleke et al., 2017), however we observed an increase of TOC in the presence of the microbial consortium (**Fig. 1**)."

L304: JA is typically involved in defense against herbivores, not microbes. there you would look into salicylic acid.

We agree with the reviewer therefore, we removed the sentence because there were no herbivores in our system.

Figures: it needs to be stated in all legends that this data is based on pooled data or on technical replicates (TOC).

We agree with the reviewer. Figure 1 has the statement that the data is based on technical replications. Figure 2 and 3 has the statements that the data is based on pooled samples.

Figure 1: please indicate number of replicates, and please highlight that these are technical replicates.

We agree with the reviewer; Figure 1 states data is based on three technical replications.

Figure 1: please add TOC measurements of negative controls. How much above background are the measurements?

We agree that negative control measurements should be included in the manuscript. However, given that negative control concentrations were below -0.56, we included the negative controls in the legend so as not to skew the y-axis (Line 173). Additionally, we have included the negative control measurements in the methods description of the TOC data collection (Lines 248 to 249).

Table 1: please provide the raw values of the phenotypic data. I am specicifally interested in seeing how much root mass was there.

We added Supplementary Table 2, "Raw values for plant metrics."

DECISION LETTER- ROUND 3

October 13, 2023
Dr. Tomas Allen Rush
Oak Ridge National Laboratory
Bioscience Divsion
1 Bethel Valley Road
Oak Ridge, Tennessee 37831-2008

MSID: 2023-01203R2
MS TITLE: Interactions with microbial consortia have variable effects in organic carbon and production of exometabolites among genotypes of Populus trichocarpa

Dear Dr. Tomas Rush:

I am pleased to inform you that your manuscript "Interactions with microbial consortia have variable effects in organic carbon and production of exometabolites among genotypes of Populus trichocarpa" has been accepted for publication in Plant Direct.

Your article will appear online in the next available issue of Plant Direct. To ensure your article gets published as quickly as possible, please pay attention to the steps detailed below. We have found that most of the delays happen at this stage, especially at the payment stage, so please respond as

quickly as possible when prompted.

License Agreement: Once your article has been accepted it will move to Production and undergo admin and file checking - you may receive an email with any queries we have at this stage. When all required items are received by the publisher and queries resolved, the corresponding author will receive an email from Wiley's Author Services system which will ask them to log on at https://authorservices.wiley.com/bauthor and will present them with the appropriate license for completion. Your article cannot be published until both the signed license agreement and payment of the article fee have been received.

Payment of the Open Access Article Publication Fee: All articles published in Plant Direct are fully open access: immediately and freely available to read, download and share. Plant Direct charges a publication fee to cover the publication costs. The corresponding author for this manuscript should have already received a quote from the payments team (cs-openaccess@wiley.com) with the estimated article publication fee; please email cs-openaccess@wiley.com if this has not been received. The corresponding author should log on to the Wiley Author Services site, where the publication fee can be paid by credit card or an invoice. Pro Forma can also be requested. Payment of the publication charge must be received before the article will be published online.

Proofs: You will have the opportunity to look over your paper once more when you receive the author proofs for your article. The proofs will be with you in approximately two weeks. Please note that, in addition to publishing reviewer comments, the author's responses to review comments will also be published alongside the final version of the paper. If you would not like the author's responses to be published, please contact the editorial office at plantdirect@wiley.com .

Promotion of your article: You can help your research get the attention it deserves! Check out Wiley's free Promotion Guide for best-practice recommendations for promoting your work at www.wileyauthors.com/eeo/guide. And learn more about Wiley Editing Services which offers professional video, design, and writing services to create shareable video abstracts, infographics, conference posters, lay summaries, and research news stories for your research at www.wileyauthors.com/eeo/promotion.

Thank you again for your contribution to Plant Direct. If you have any questions, feel free to contact the editorial office at plantdirect@wiley.com .

Sincerely,

Hannah Schneider

Gustavo MacIntosh

Editor, Plant Direct

-- Editor comments

-- Reviewer comments:

AUTHORS' RESPONSE

We are very grateful for the time that the editor and reviewer spent reading our manuscript and the very valuable comments and suggestions. We have carefully considered the recommendations and addressed all of them to improve the manuscript.

Please find below a detailed point-by-point response to all comments. The reviewer comments are in black, and our responses are in blue. We also provide a revised version of the manuscript in which all modifications have been highlighted ("Tracked Changes Carrell et al, - Manuscript Revised v2").

Please note that the line numbers that we refer to below are for the "Tracked Changes Carrell et al, - Manuscript Revised v2"

Editor comments:

Thank you for your revisions. Please consider making some statements and conclusions in the manuscript a bit more subtle to reflect the limited number of genotypes and replications in the study. For example, in the abstract line 23 "across various poplar genotypes" could be changed to 'across four different poplar genotypes' and line 107-108 'on plant growth. However they did produce changes in the root...' could be changed to 'on plant growth in four poplar genotypes. However, they did produce changes in the root exudate exometabolomes, which results suggested was plant host genotype dependent'.

We agree and made the changes the editor had suggested on lines 23; and 107 to 108.

Reviewer #1 comments:

thank you for your responses.

Thank you.
